# Trifecta Outcomes in Open, Laparoscopy or Robotic Partial Nephrectomy: Does the Surgical Approach Matter?

**DOI:** 10.15586/jkcvhl.2019.115

**Published:** 2019-05-13

**Authors:** Ketan Mehra, Ramanitharan Manikandan, Lalgudi Narayanan Dorairajan, Sreenivasan Sreerag, Amit Jain, Sri Harsha Bokka

**Affiliations:** Department of Urology, Jawaharlal Institute of Postgraduate Medical Education and Research (JIPMER), Puducherry, India

**Keywords:** partial nephrectomy, renal cell carcinoma, renal tumors, robotic partial nephrectomy, trifecta outcomes

## Abstract

This retrospective study evaluated perioperative outcomes of open partial nephrectomy (OPN), laparoscopic partial nephrectomy (LPN), and robot-assisted partial nephrectomy (RAPN) and identified predictive factors of Trifecta achievement for renal tumors that underwent partial nephrectomy (PN) in a single institutional cohort. The study involved patients who underwent PN from January 2011 to July 2018. Trifecta was defined as absence of perioperative complications, no positive surgical margins, and ischemia time <30 min. Fifty-five PN procedures were reviewed: 28 OPN, 14 LPN, and 13 RAPN. OPN, LPN and RAPN had similar median tumor size (5.75, 5.25, and 5 cm), nephrometry score (7, 6, and 6), and preoperative creatinine (1.09, 1.1, and 1.1 mg/dl, respectively). Blood loss was higher for OPN (550 ml) than for LPN (400 ml) and RAPN (300 ml), P = 0.042. Drain was removed after 6 days in OPN which was higher than LPN and RAPN (4.5 and 4 days, respectively), P = 0.008. OPN, LPN, and RAPN had similar median operative time (190, 180, and 180 min, respectively), P = 0.438. Median postoperative stay for OPN, LPN, and RAPN was 5, 6.5, and 10 days, respectively. Trifecta outcomes of 73.1%, 64.3%, and 61.53% were achieved in OPN, LPN, and RAPN, respectively, P = 0.730. It was concluded that Trifecta outcomes had no significant difference among OPN, LPN, and RAPN. LPN can produce as good results as RAPN. Keeping in mind the cost-effectiveness, LPN holds an important position in developing countries where expenditure by patient is a major factor.

## Introduction

Currently, partial nephrectomy (PN) is the preferred modality of treatment for small renal masses. The American Urological Association Guideline recommends nephron-sparing surgery for T1 renal mass, as there is increased risk of chronic kidney disease (CKD) associated with radical nephrectomy (RN) (1). Furthermore, European Association of Urology (EAU) guidelines have also recommended PN for T1b tumors as it preserves well normal renal parenchyma as well as provides oncological efficacy (2). Open PN (OPN) has been considered as the “gold standard” approach for many years. With advancements in laparoscopic techniques, equipment, and surgeons’ skills, laparoscopy has been adopted worldwide, thereby offering comparable oncological outcomes, less morbidity, and shortened convalescence compared to open approach (2–7). But laparoscopic PN is a technically challenging procedure which is presently limited to few surgeons in select centers in developing countries. Since the last decade, with widespread diffusion of robotic technology, there is increased adoption of robotic-assisted partial nephrectomy in providing a minimally invasive option for patients with clinical T1a lesions.

The outcomes of PN have been defined in terms of “Trifecta” which means no complications, negative surgical margins, and minimal renal functional decrease (8). The objective of the present study is to determine the complications and perioperative outcome measures associated with different techniques of PN for renal tumors.

## Materials and Methods

After taking institutional ethics approval, the records of patients who underwent OPN, LPN, and RAPN for renal masses at the Jawaharlal Institute of Postgraduate Medical Education and Research (JIPMER), Puducherry, between January 2011 and July 2018 were reviewed. Patients having incomplete data, solitary kidneys, clinical evidence of metastatic renal cancer, previous renal carcinoma, or renal surgery were excluded.

For each RAPN, two OPNs were matched in terms of patient age (within 10 years) and RENAL nephrometry score (within 2) from a pool of all PN cases performed between January 2011 and July 2018. RAPN and LPN cases were not directly matched because they were performed by the same two surgeons with equal number of cases and the same mass characteristics were used to select patients for both of these treatment modalities. No significant difference was seen in the mass size or nephrometry score between the two groups. The preoperative characteristics of these two groups were compared to ensure that they were similar.

The baseline demographic, disease, and treatment-related parameters were extracted from the hospital records in a de-identified manner. These data included age, sex, size, and location of tumor, mode of surgery (OPN/LPN/RAPN), indication for surgery, tumor pathology, serum creatinine preoperatively and at discharge. Perioperative data included duration of surgery, blood loss, number of blood transfusions, warm ischemia time (WIT), day of drain removal, hospital stay, need for intraoperative cooling, and postoperative complications. Nephrometry score was determined using the formula by Kutikov and Uzzo (9). The margin status of the resected tumor was recorded from the final histopathological examination report. The preoperative characteristics of the groups were compared to ensure that they were similar. The Trifecta achieved was defined as negative surgical margins, no serious perioperative complications (Clavien Dindo >2), and WIT<30 min.

The data were analyzed using SPSS, version 19. Comparison of the medians between groups was made using the Kruskal–Wallis test. The nominal variables were compared using chi-square test or Fisher’s exact tests to determine significance.

## Results

Among the 53 patients who underwent PN, 26 had OPN, 14 had LPN, and 13 had RAPN (Table 1). The patient characteristics in OPN, LPN, and RAPN were similar in terms of median tumor size (5.75, 5.25, and 5 cm, respectively, P = 0.219), age (49 years, Range: 29–80; 48.5 years, Range:19–58; and 42 years, Range: 29–72, P = 0.338), median preoperative creatinine values (1.09, 1.1, and 1.1 mg/dl, respectively), and tumor complexities using RENAL nephrometry score as a measure (7, 6, and 7, respectively). Median blood loss was higher for OPN (500 ml, Range: 100–2,000) than LPN (400 ml, Range: 150–750) and RAPN (300 ml, Range: 100–800), P = 0.042. Median postoperative hospital stay was lower for RAPN (5 days, Range: 4–24) than OPN (10 days, Range: 6–22) and LPN (6.5 days, Range: 5–10), P = 0.154. Postoperative drain was removed after 6, 4.5, and 4 days in OPN, LPN, and RAPN, respectively, P = 0.008. OPN, LPN, and RAPN were similar in median operative time (190, 180, and 180 min, respectively, P = 0.438) and median WIT (23, 24.5, and 27 min, respectively, P = 0.923). Cold ischemia was used in 12 (46.1%) OPN while all LPN and RAPN underwent warm ischemia.

**Table 1:** Patient and tumor characteristic

Variables	OPN (n = 26)	LPN(n = 14)	RAPN (n = 13)	P
Median age, years (Range)	49 (29–80)	48.5 (19–58)	42 (29–72)	0.338
Males (%)	16 (61.5)	8 (57.1)	8 (61.5)	0.959
Females (%)	10 (38.5)	6 (42.9)	5 (38.5)	0.959
Median size, cm (Range)	5.75 (3–16)	5.25 (1.6–15)	5 (2–11)	0.219
Median preop Cr^a^, mg/dl (Range)	1.09 (0.8–4.7)	1.1 (0.8–1.8)	1.1 (0.9–5.5)	0.935
Median preop eGFR^b^, ml/min/1.73 m^2^ (Range)	64.06 (13.8–111.6)	62.21 (43–98.2)	67.06 (11.9–100.2)	0.954
Median postop Cr^a^, mg/dl (Range)	1.11 (0.7–9.1)	1.0 (0.8–2.1)	1.1 (0.73–9.09)	0.421
Median postop eGFR^b^, ml/min/1.73 m^2^ (Range)	59.20 (6.5–114.9)	62.89 (36–116.3)	65.62 (6.7–93.9)	0.401
Hypertension (%)	7 (26.9)	3 (21.4)	3 (23)	0.482
Diabetes mellitus (%)	6 (23)	3 (21.4)	2 (15.3)	0.324
Median RENAL score (Range)	7 (4–11)	6 (4–10)	7 (4–10)	0.177
Malignant tumor (%)	20 (76.9)	10 (71.4)	11 (84.6)	0.714
Clear-cell RCC (%)	18 (90)	8 (80)	9 (81.8)	
Papillary cell RCC^c^ (%)	2 (10)	1 (10)	2 (18.2)	
Multilocular cystic carcinoma (%)	-	1 (10)	-	
Benign tumors (%)	6 (23)	4 (28.5)	2 (15.4)	
AML^d^ (%)	3 (50)	2 (50)	2 (100)	
MEST^e^ (%)	1 (16.6)	-	-	
Infectious (%)	2 (33.3)	2 (50)	-	

a Creatinine.

b Glomerular filtration rate.

c Renal cell carcinoma.

d Angiomyolipoma.

e Mixed epidermal stromal tumor.

The postoperative complications (Clavien Dindo >2) were two in each group (Table 2). It comprised four urinoma (one in OPN, two in LPN, and one in RAPN) which was drained by putting a pigtail and Foley’s catheter. One patient of RAPN developed pseudoaneurysm and underwent angioembolisation. One patient of OPN had to be managed in the intensive care unit due to sepsis. Total number of patients requiring blood transfusions in OPN, LPN, and RAPN were 6 (23%), 1 (7%), and 1(7%), respectively, P = 0.281. OPN, LPN, and RAPN had one case each with positive surgical margins on histopathological report. Malignancies were identified in 20 (76.9%) OPN, 10 (71.4%) LPN, and 11 (84.6%) RAPN (Table 1); the majority were clear-cell carcinoma. One patient of LPN postoperative was identified as renal cell carcinoma with Fuhrman grade 3 with renal sinus involvement and underwent radical nephrectomy later. Trifecta was achieved in 19 (73.1%) in OPN, 9 (64.3%) in LPN, and 8 (61.5%) in RAPN, P = 0.730.

**Table 2:** Complications and TRIFECTA

Variables (no. of patients)		OPN (26)	LPN (14)	RAPN (13)	P
Postop Complications		2	2	2	--
Grade 3		1	2	1	--
Grade 4		1	-	1	--
Total patients with blood transfusion (%)		6 (23)	1(7)	1(7)	0.281
Urine leak/Urinoma		1	2	1	--
Post op proceeded for radical nephrectomy		--	1	--	--
Positive surgical margins		1	1	1	--
Trifecta	Achieved (%)	19 (73.1)	9 (64.3)	8 (61.53)	0.730
	Not achieved (%)	7 (26.9)	5 (35.7)	5 (38.47)	

## Discussion

In our study, 53 PN (26 OPN, 14 LPN, and 13 OPN) were reviewed with similar tumor size, nephrometry score, age, and gender. OPN had significantly higher blood loss than LPN, which in turn was higher than RAPN. Postoperative days for drain removal were significantly lower in RAPN and LPN than OPN. WIT was similar in all the three groups. In OPN, LPN, and RAPN, postoperative significant complications (Clavien grade>2) were 7.6%, 14.2%, and 15.3%, respectively, and negative surgical margins were 3.8%, 7.1%, and 7.6%, respectively, but the differences were not significant.

Many studies in the past have compared RAPN with LPN and OPN with LPN. Both RAPN and LPN offer equal success with low morbidity. Benway et al. in a multi-institutional study compared RAPN (129 cases) and LPN (118 cases) (10). Both RAPN and LPN had similar postoperative complications including urine leak (3 and 4, respectively) and hemorrhage (2 and 1, respectively). WIT was shorter for RAPN than for LPN (19.7 min vs 28.4 min). Similar to our study, blood loss was significantly lower in RAPN (155 ml vs 196 ml). Two other single institutional studies by Wand et al. and Kural et al. suggested no difference in blood loss, complications, and margin status, but WIT was lower with RAPN (11, 12). Contradictory to the abovementioned studies, Haber et al. compared 75 RAPN with 75 LPN and noted no significant difference in WIT (18.2 min in RAPN vs 20.3 min in LPN) (13). There was significantly higher blood loss in RAPN (323 ml) versus LPN (222 ml). There was a decrease in postoperative glomerular filtration rate (GFR) in 9% patients in both the groups, and 13% RAPN and 16% LPN had complications. To summarize, RAPN and LPN have same perioperative morbidity in literature. But these series do not account for location of tumor or complexity.

Comparison of OPN to RAPN is limited in literature. Several studies have compared OPN to LPN. Gill et al. compared 1,028 OPN with 771 LPN and showed reduced WIT for OPN (30.7 min) versus LPN (20.1 min) (14). Similar to our study, postoperative complications and positive margins (1.6% OPN vs 2.85% LPN) were similar. In contrast to our study, blood loss was similar for OPN (300 ml) and LPN (376 ml), with 5% blood transfusion rate in both the groups. Further the size and configuration of the tumors significantly differed as OPN had larger tumors, which were more endophytic.

Pempangkosol et al. compared 58 OPN (median size 2.9 cm) with 85 LPN (2.4 cm) (15). In contrast to our study, the overall complications were 25% in OPN and 7.8% in LPN. Each group had three intraoperative complications, one urine leak/urinoma, and two postoperative bleeding that required blood transfusion.

In another study done by Marszalek et al., cases were matched by size and configuration (16). They compared 100 OPN with 100 LPN. They reported shorter median operative time, more frequent intraoperative complications (10% vs 3%), and less postoperative complications (14% vs 19%) in LPN. In our study, OPN and RAPN groups had similar median operative time (190 vs 180 min) and WIT (23 vs 27 min) while blood loss was less for RAPN than OPN (300 ml vs 550 ml). Comparison of WIT is limited because cold ischemia time for OPN was not included. Margin status, postoperative renal function, and complications were similar.

Lucas et al. did a matched comparison in terms of tumor configuration with the help of nephrometry score, tumor size, gender, age and compared 54 OPN, 15 LPN, and 27 RAPN (17). There was similar distribution of low, medium, and high complex lesions in OPN and RAPN while LPN had no high complexity lesions. OPN had lower operative time and ischemia time but increased blood loss. Postoperatively all groups had lower urine leaks (3.7% RAPN, 0 LPN, and 5.6% OPN), with similar GFR. Tumor at the specimen margin was also low (3.7% RAPN, 0 LPN, and 7.4% OPN). There are some other studies comparing Trifecta outcomes in RAPN with LPN (Table 3).

**Table 3:** TRIFECTA in other studies

Studies	Year	Number	Trifecta achieved (%)
Khalifeh et al (18)	2013	500	RAPN^a^-58 LPN^b^-31
Lista et al. (19)	2015	339	RAPN-67
Zargar et al. (20)	2015	1831	RAPN-70 LPN-33
Carneiro et al. (21)	2015	347	LPN-48 RAPN-81
Our Study	2018	53	OPN^c^-73 LPN-64 RAPN-62

a Robotic-assisted partial nephrectomy.

b Laparoscopic-assisted partial nephrectomy.

c Open partial nephrectomy.

There are limitations to our study. Firstly, the sample size was small compared to earlier studies. While we identified statistically significant differences between different treatment modalities, the study was not adequately powered for detecting subtle differences. Secondly, LPN and RAPN cases were not matched due to constraints of small cohorts and as the same two surgeons performed both LPN and RAPN cases in equal numbers it was assumed that LPN cases would be similar to RAPN. Analysis reveals that tumors were almost similar in all three groups in all aspects. The third limitation is that the surgeries were performed by four different surgeons, resulting in differences in total operating time, WIT, and outcomes due to differences in surgical technique. A final limitation was that all the RAPN performed were the initial robotic surgeries performed at our institution and hence the surgeons were in their initial learning curve affecting the total operating time and other outcomes.

## Conclusion

Renal tumors can be safely treated by LPN or RAPN with lesser morbidity as compared to OPN. Trifecta outcome had no significant difference among OPN, LPN, and RAPN. LPN can produce as good results as RAPN. Keeping in mind the cost-effectiveness, LPN holds an important position in developing countries where expenditure by patient is a major factor. It stands equal to open surgeries in terms of surgical and oncological outcomes, with significantly lesser morbidity than the open procedures.

## Conflicts of Interest

The authors declare no potential conflicts of interest with respect to research, authorship, and publication of this article.

## References

[1] Almassi N, Gill BC, Rini B, Fareed K Management of small renal masses. Transl Androl Urol. 2017 10;6(5):923–30. 10.21037/tau.2017.07.11 29184793 PMC5673824

[2] Ljungberg B, Bensalah K, Canfield S, Dabestani S, Hofmann F, Hora M, et al. EAU guidelines on renal cell carcinoma: 2014 update. Eur Urol. 2015 5;67(5):913–24. 10.1016/j.eururo.2015.01.005 25616710

[3] Badalato GM, Kates M, Wisnivesky JP, Choudhury AR, McKiernan JM Survival after partial and radical nephrectomy for the treatment of stage T1bN0M0 renal cell carcinoma (RCC) in the USA: a propensity scoring approach. BJU Int. 2012 5;109:1457–62. 10.1111/j.1464-410X.2011.10597.x 21933334

[4] Mitchell RE, Gilbert SM, Murphy AM, Olsson CA, Benson MC, McKiernan JM Partial nephrectomy and radical nephrectomy offer similar cancer outcomes in renal cortical tumors 4 cm or larger. Urology. 2006 2;67:260–4. 10.1016/j.urology.2005.08.057 16461075

[5] Thompson RH, Siddiqui S, Lohse CM, Leibovich BC, Russo P, Blute ML Partial versus radical nephrectomy for 4 to 7 cm renal cortical tumors. J Urol. 2009 12;182:2601–6. 10.1016/j.juro.2009.08.087 19836797 PMC4171846

[6] Weight CJ, Larson BT, Gao T, Campbell SC, Lane BR, Kaouk JH, et al. Elective partial nephrectomy in patients with clinical T1b renal tumors is associated with improved overall survival. Urology. 2010 9;76:631–7. 10.1016/j.urology.2009.11.087 20451967

[7] Porpiglia F, Mari A, Bertolo R, Antonelli A, Bianchi G, Fidanza F, et al. Partial Nephrectomy in Clinical T1b Renal Tumors: Multicenter Comparative Study of Open, Laparoscopic and Robot-assisted Approach (the RECORd Project). Urology. 2016 3;89:45–51. 10.1016/j.urology.2015.08.049 26743388

[8] Hung AJ, Cai J, Simmons MN, Gill IS “Trifecta” in partial nephrectomy. J Urol. 2013 1;189(1):36–42. 10.1016/j.juro.2012.09.042 23164381

[9] Kutikov A, Uzzo RG The R.E.N.A.L. nephrometry score: a comprehensive standardized system for quantitating renal tumor size, location and depth. J Urol. 2009 9;182(3):844–53. 10.1016/j.juro.2009.05.035 19616235

[10] Benway BM, Bhayani SB, Rogers CG, Dulabon LM, Patel MN, Lipkin M, et al. Robot assisted partial nephrectomy versus laparoscopic partial nephrectomy for renal tumors: a multi-institutional analysis of perioperative outcomes. J Urol. 2009 9;182(3):866–72. 10.1016/j.juro.2009.05.037 19616229

[11] Wang AJ, Bhayani SB Robotic partial nephrectomy versus laparoscopic partial nephrectomy for renal cell carcinoma: single-surgeon analysis of >100 consecutive procedures. Urology. 2009 2;73(2):306–10. 10.1016/j.urology.2008.09.049 19038419

[12] Kural AR, Atug F, Tufek I, Akpinar H Robot-assisted partial nephrectomy versus laparoscopic partial nephrectomy: comparison of outcomes. J Endourol. 2009 9;23(9):1491–7. 10.1089/end.2009.0377 19694519

[13] Haber GP, White WM, Crouzet S, White MA, Forest S, Autorino R, et al. Robotic versus laparoscopic partial nephrectomy: single-surgeon matched cohort study of 150 patients. Urology. 2010 9;76(3):754–8. 10.1016/j.urology.2010.03.058 20646744

[14] Gill IS, Kavoussi LR, Lane BR, Blute ML, Babineau D, Colombo JR Jr, et al. Comparison of 1,800 laparoscopic and open partial nephrectomies for single renal tumors. J Urol. 2007 7;178(1):41–6. 10.1016/j.juro.2007.03.038 17574056

[15] Permpongkosol S, Bagga HS, Romero FR, Sroka M, Jarrett TW, Kavoussi LR Laparoscopic versus open partial nephrectomy for the treatment of pathological T1N0M0 renal cell carcinoma: a 5-year survival rate. J Urol. 2006 11;176(5):1984–8. 10.1016/j.juro.2006.07.033 17070227

[16] Marszalek M, Meixl H, Polajnar M, Rauchenwald M, Jeschke K, Madersbacher S Laparoscopic and open partial nephrectomy: a matched-pair comparison of 200 patients. Eur Urol. 2009 5;55(5):1171–8. 10.1016/j.eururo.2009.01.042 19232819

[17] Lucas SM, Matthew MJ, Erntsberger L, Sundaram CP A Comparison of Robotic, Laparoscopic and Open Partial Nephrectomy. JSLS. 2012 Oct-Dec;16:581–7. 10.4293/108680812X13462882737177 23484568 PMC3558896

[18] Khalifeh A, Autorino R, Hillyer SP, Laydner H, Eyraud R, Panumatrassamee K, et al. Comparative out comes and assessment of trifecta in 500 robotic and laparoscopic partial nephrectomy cases: a single surgeon experience. J Urol. 2013 4;189(4):1236–42. 10.1016/j.juro.2012.10.021 23079376

[19] Lista G, Buffi NM, Lughezzani G, Lazzeri M, Abrate A, Mistretta A, et al, Margin, ischemia, and complications system to report perioperative outcomes of robotic partial nephrectomy: A European Multicenter Observational Study (EMOS project). Urology. 2015 3;85(3):589–95. 10.1016/j.urology.2014.09.068 25733270

[20] Zargar H, Allaf ME, Bhayani S, Stifelman M, Rogers C, Ball MW, et al., Trifecta and optimal perioperative outcomes of robotic and laparoscopic partial nephrectomy in surgical treatment of small renal masses: A multi-Institutional study. BJU Int. 2015 9;116(3):407–14. 10.1111/bju.12933 25220543

[21] Carneiro A, Sivaraman A, Sanchez-Salas R, Di Trapani E, Barret E, Rozet F, et al. Evolution from laparoscopic to robotic nephron sparing surgery: A high-volume laparoscopic center experience on achieving “trifecta” outcomes. World J Urol. 2015 12;33(12):2039–44. 10.1007/s00345-015-1552-1 25869814

